# The genome sequence of the brown sea anemone, *Metridium senile *(Linnaeus, 1761)

**DOI:** 10.12688/wellcomeopenres.20376.1

**Published:** 2023-11-20

**Authors:** Patrick Adkins, John Bishop, Rob Mrowicki, Mark L. Blaxter, Vengamanaidu Modepalli, Marine Biological Association Genome Acquisition Lab, Darwin Tree of Life Barcoding collective, Wellcome Sanger Institute Tree of Life programme, Wellcome Sanger Institute Scientific Operations: DNA Pipelines collective, Tree of Life Core Informatics collective, Darwin Tree of Life Consortium

**Affiliations:** 1 The Marine Biological Association, Plymouth, England, UK; 2 Tree of Life, Wellcome Sanger Institute, Hinxton, England, UK

**Keywords:** Metridium senile, brown sea anemone, genome sequence, chromosomal, Actiniaria

## Abstract

We present a genome assembly from an individual *Metridium senile* (the brown sea anemone; Cnidaria; Anthozoa; Actiniaria; Metridiidae). The genome sequence is 390.9 megabases in span. Most of the assembly is scaffolded into 16 chromosomal pseudomolecules. The mitochondrial genome has also been assembled and is 17.44 kilobases in length.

## Species taxonomy

Eukaryota; Metazoa; Eumetazoa; Cnidaria; Anthozoa; Hexacorallia; Actiniaria; Nynantheae; Metridiidae; *Metridium*; *Metridium senile* (Linnaeus, 1761) (NCBI:txid6116).

## Background

*Metridium senile*, the frilled or plumose sea anemone, is among the largest sea anemones, reaching up to one metre in height ( Stephenson, 1928). Sea anemones, corals, jellyfish, and hydras constitute the oldest eumetazoan phylum, the Cnidaria. The class Anthozoa encompasses sea anemones (Actinaria) and reef-building scleractinian corals. Molecular phylogenetic analyses suggest that corals and sea anemones diverged approximately 500 million years ago (the late Cambrian or early Ordovician) ( Shinzato *et al.*, 2011). *Metridium* species are commonly found in coastal waters across the Northern Hemisphere, at depths ranging from the shallows to depths of up to 100 metres.

*Metridium senile* exhibits both sexual and asexual reproduction through pedal laceration ( Glon *et al.*, 2020; Hoffmann, 1976). The species is an oviparous broadcast spawner, undergoing external fertilisation upon release of gametes in late summer to early fall to produce free-swimming planula larvae ( Lombardi & Lesser, 2010). The larva spends several months in the planktonic form before settling into a juvenile polyp ( Sebens, 1981).

*Metridium senile* prevalence and distribution have made it a familiar sight to many marine enthusiasts and researchers alike ( Glon *et al.*, 2020). *Metridium senile* ( Linné, 1761) was first described from Scandinavia and has since become well-documented in various regions, including the British Isles, northern Europe, and North America’s Atlantic and Pacific coasts ( Verrill, 1865; Verrill, 1870). *Metridium* was one of the first anemone species to be the subject of genetic research due to its accessibility as intertidal species and the known diversity in their body size, colour, and biotic processes as incidence or asexual reproduction ( Bucklin & Hedgecock, 1982). The taxonomic history of the species has been challenging, as there has been long-term confusion about how to identify and apply the names of *Metridium senile* and *Metridium dianthus* (Ellis, 1768) of the north-eastern Atlantic. The name *Metridium senile* has been used for populations on both the north-western and north-eastern Atlantic coasts and for populations in the North Pacific ( Glon *et al.*, 2020). Earlier biochemical genetic studies have corroborated morphological evidence that European populations constitute the subspecies *M. s. senile* (Linnaeus). North-western Atlantic representatives belong to the subspecies *M. s. marginatum* (Le Sueur), and southwestern Atlantic ones to *M. s. lobatum* (Carlgren). Furthermore, *Metridium senile* was classified into two distinct clusters, one containing clonal and one containing solitary individuals, with the large and solitary form later being raised to species level as *Metridium farcimen* ( Brandt, 1834; Fautin *et al.*, 1989). Of the six putative species in the genus *Metridium*, *M. senile* has the broadest range, with a circumboreal distribution that overlaps the distribution of nearly every other species in the genus ( Fautin, 2016; Stephenson, 1928). Its high intra-population variation in anatomy, reproduction, and genetics, along with its geographic distribution, make the genome of *Metridium senile* an effective system in investigating the connection of genetic and morphological diversity.

## Genome sequence report

**Figure 1.**
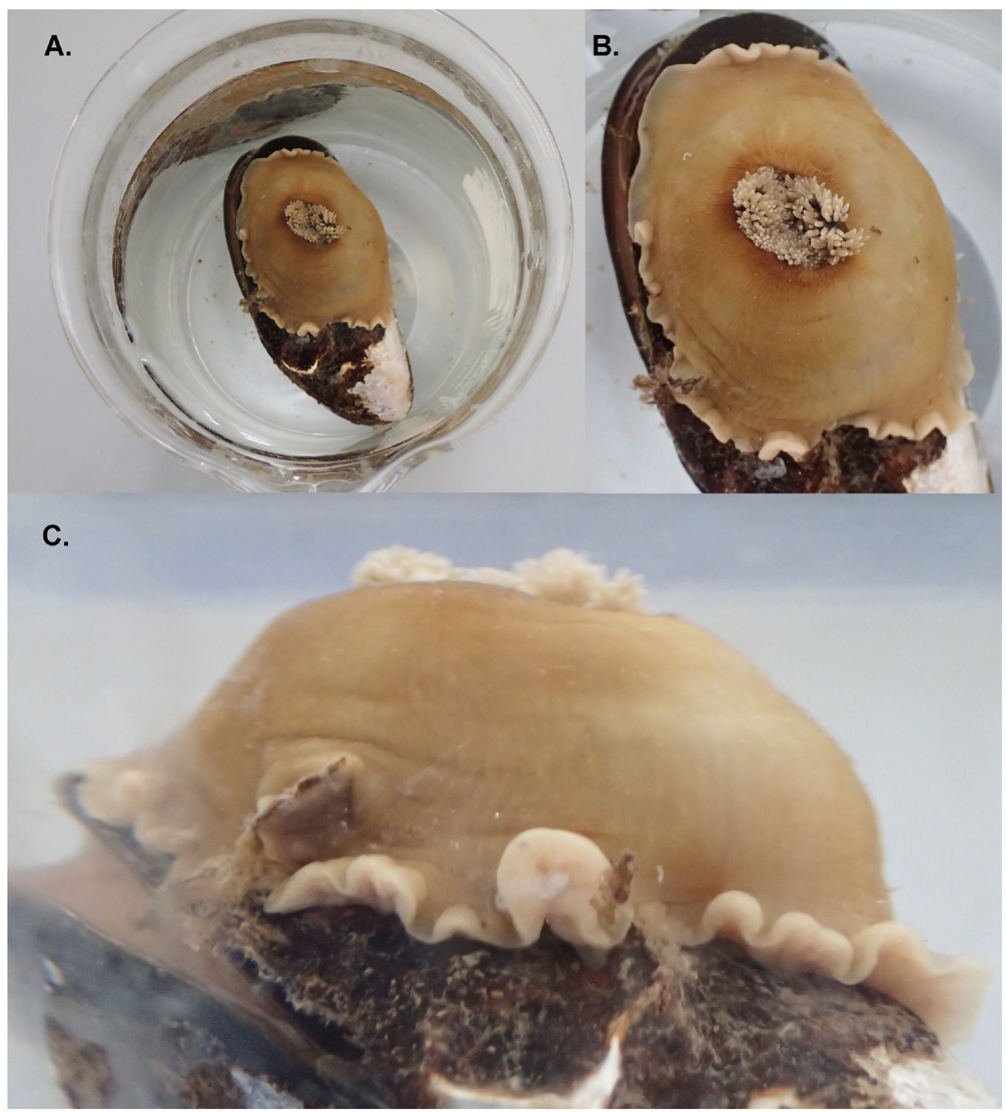
Photographs of the *Metridium senile* (jaMetSeni4) specimen used for genome sequencing showing dorsal views of the retracted animal ( **A** and **B**), and a lateral view of the column of the retracted animal ( **C**).

The genome was sequenced one *Metridium senile* ( Figure 1) collected from Queen Anne’s Battery Marina visitors’ pontoon, Plymouth, UK (50.36, –4.13). A total of 59-fold coverage in Pacific Biosciences single-molecule HiFi long reads was generated. Primary assembly contigs were scaffolded with chromosome conformation Hi-C data. Manual assembly curation corrected 137 missing joins or mis-joins and removed 33 haplotypic duplications, reducing the assembly length by 1.44% and the scaffold number by 13.45%, and increasing the scaffold N50 by 0.86%.

**Figure 2.**
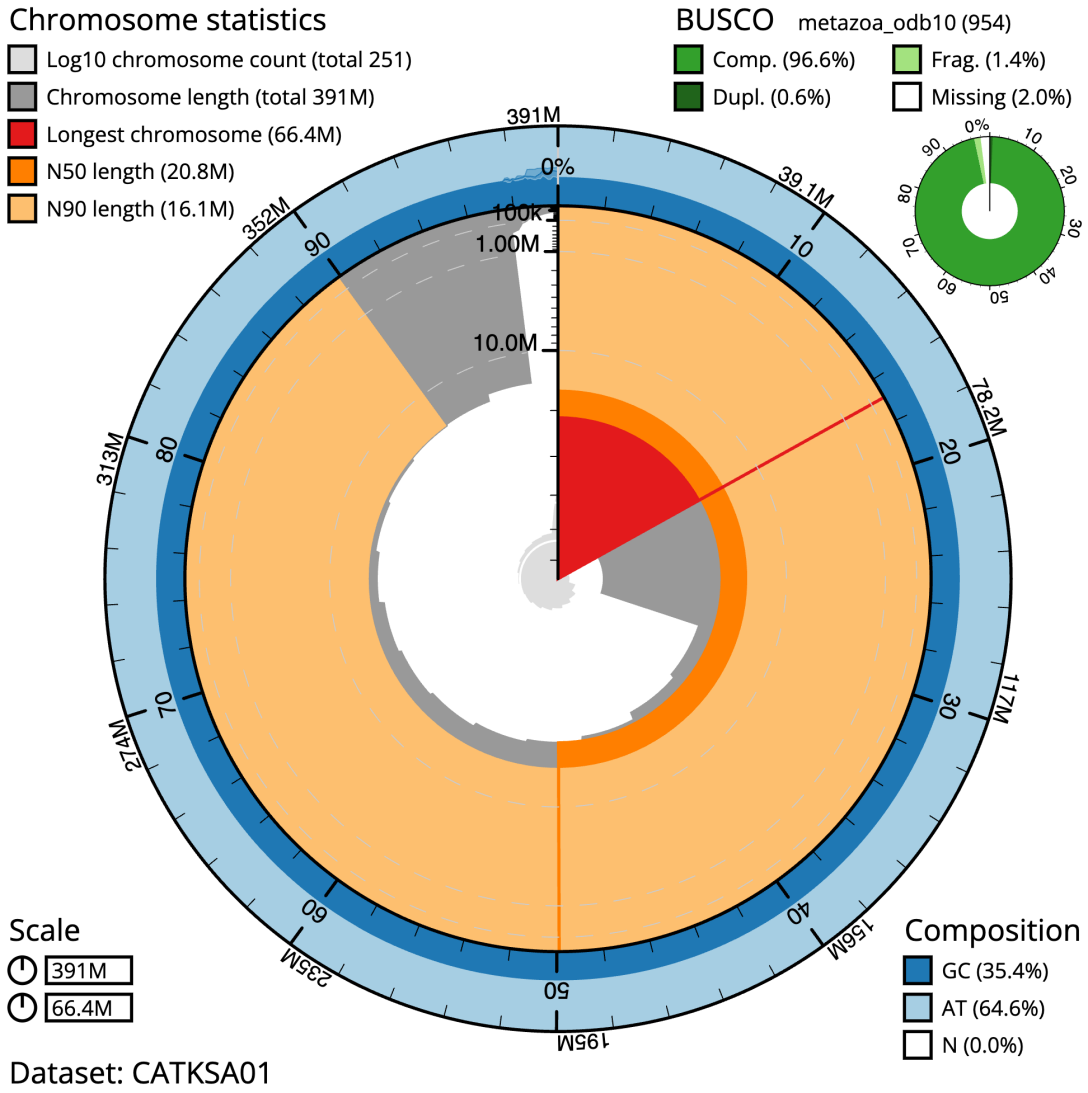
Genome assembly of *Metridium senile*, jaMetSeni4.1: metrics. The BlobToolKit Snailplot shows N50 metrics and BUSCO gene completeness. The main plot is divided into 1,000 size-ordered bins around the circumference with each bin representing 0.1% of the 390,902,175 bp assembly. The distribution of scaffold lengths is shown in dark grey with the plot radius scaled to the longest scaffold present in the assembly (66,427,095 bp, shown in red). Orange and pale-orange arcs show the N50 and N90 scaffold lengths (20,827,098 and 16,127,631 bp), respectively. The pale grey spiral shows the cumulative scaffold count on a log scale with white scale lines showing successive orders of magnitude. The blue and pale-blue area around the outside of the plot shows the distribution of GC, AT and N percentages in the same bins as the inner plot. A summary of complete, fragmented, duplicated and missing BUSCO genes in the metazoa_odb10 set is shown in the top right. An interactive version of this figure is available at https://blobtoolkit.genomehubs.org/view/Metridium%20senile/dataset/CATKSA01/snail.

The final assembly has a total length of 390.9 Mb in 250 sequence scaffolds with a scaffold N50 of 20.8 Mb ( Table 1). Most (97.85%) of the assembly sequence was assigned to 16 chromosomal-level scaffolds. Chromosome-scale scaffolds confirmed by the Hi-C data are named in order of size ( Figure 2– Figure 5; Table 2). While not fully phased, the assembly deposited is of one haplotype. Contigs corresponding to the second haplotype have also been deposited. The mitochondrial genome was also assembled and can be found as a contig within the multifasta file of the genome submission.

**Table 1.** Genome data for *Metridium senile*, jaMetSeni4.1.

Project accession data		
Assembly identifier	jaMetSeni4.1	
Assembly release date	2023-04-08	
Species	*Metridium senile*	
Specimen	jaMetSeni4	
NCBI taxonomy ID	6116	
BioProject	PRJEB59155	
BioSample ID	SAMEA110449715	
Isolate information	jaMetSeni4: DNA sequencing jaMetSeni1: Hi-C scaffolding	
Assembly metrics *		*Benchmark*
Consensus quality (QV)	55.0	*≥ 50*
*k*-mer completeness	99.99%	*≥ 95%*
BUSCO **	C:96.6%[S:96.0%,D:0.6%],F:1.4%,M:2.0%,n:954	*C ≥ 95%*
Percentage of assembly mapped to chromosomes	99.33%	*≥ 95%*
Sex chromosomes	-	*localised homologous pairs*
Organelles	Mitochondrial genome assembled	*complete single alleles*
Raw data accessions		
PacificBiosciences SEQUEL II	ERR10809397	
Hi-C Illumina	ERR10802478	
Genome assembly		
Assembly accession	GCA_949775045.1	
*Accession of alternate haplotype*	GCA_949775035.1	
Span (Mb)	390.9	
Number of contigs	590	
Contig N50 length (Mb)	1.8	
Number of scaffolds	250	
Scaffold N50 length (Mb)	20.8	
Longest scaffold (Mb)	66.4	

* Assembly metric benchmarks are adapted from column VGP-2020 of “Table 1: Proposed standards and metrics for defining genome assembly quality” from ( Rhie *et al.*, 2021). ** BUSCO scores based on the metazoa_odb10 BUSCO set using v5.3.2. C = complete [S = single copy, D = duplicated], F = fragmented, M = missing, n = number of orthologues in comparison. A full set of BUSCO scores is available at https://blobtoolkit.genomehubs.org/view/Metridium%20senile/dataset/CATKSA01/busco.

**Figure 3.**
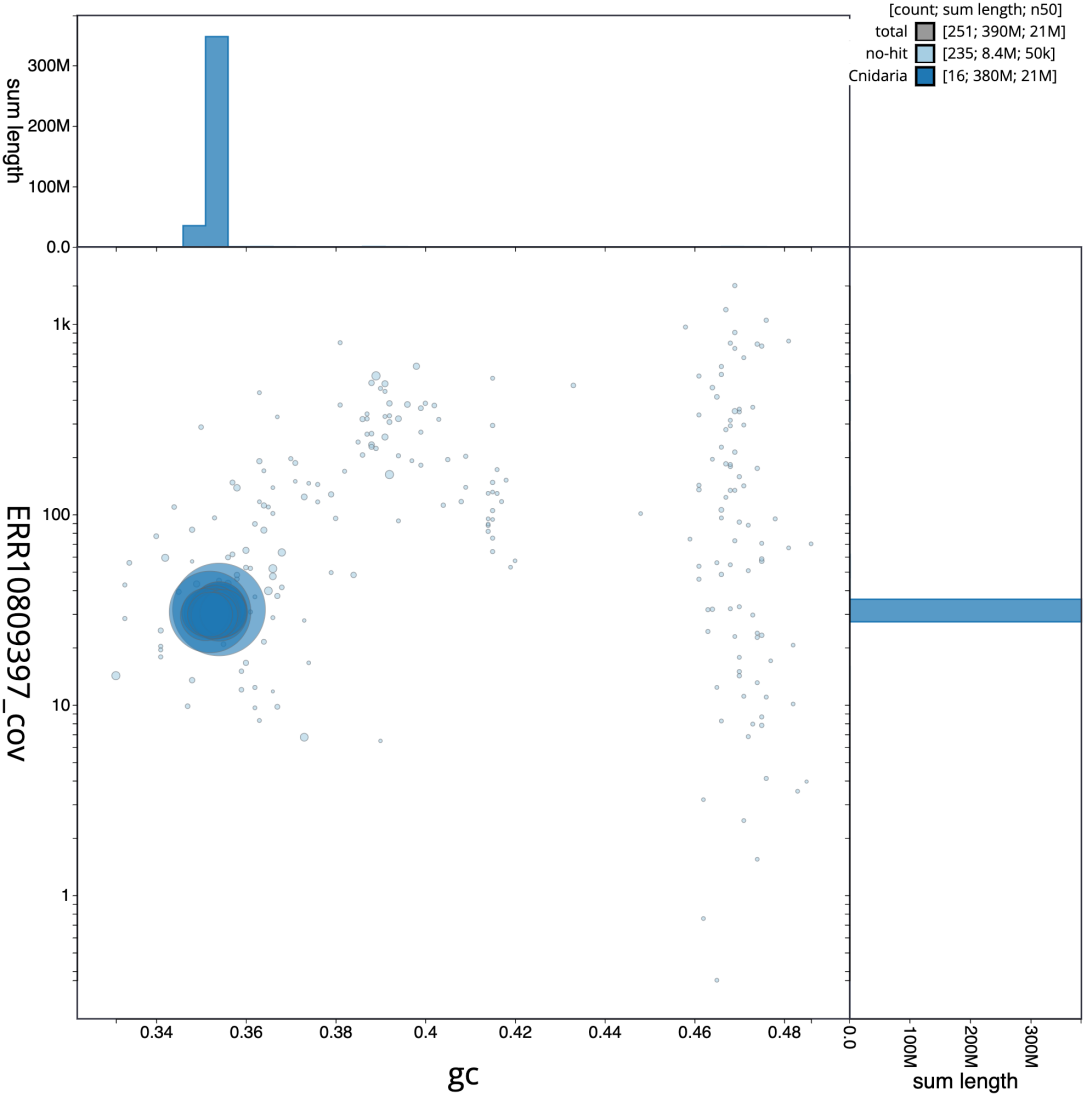
Genome assembly of *Metridium senile*, jaMetSeni4.1: BlobToolKit GC-coverage plot. Scaffolds are coloured by phylum. Circles are sized in proportion to scaffold length. Histograms show the distribution of scaffold length sum along each axis. An interactive version of this figure is available at https://blobtoolkit.genomehubs.org/view/Metridium%20senile/dataset/CATKSA01/blob.

**Figure 4.**
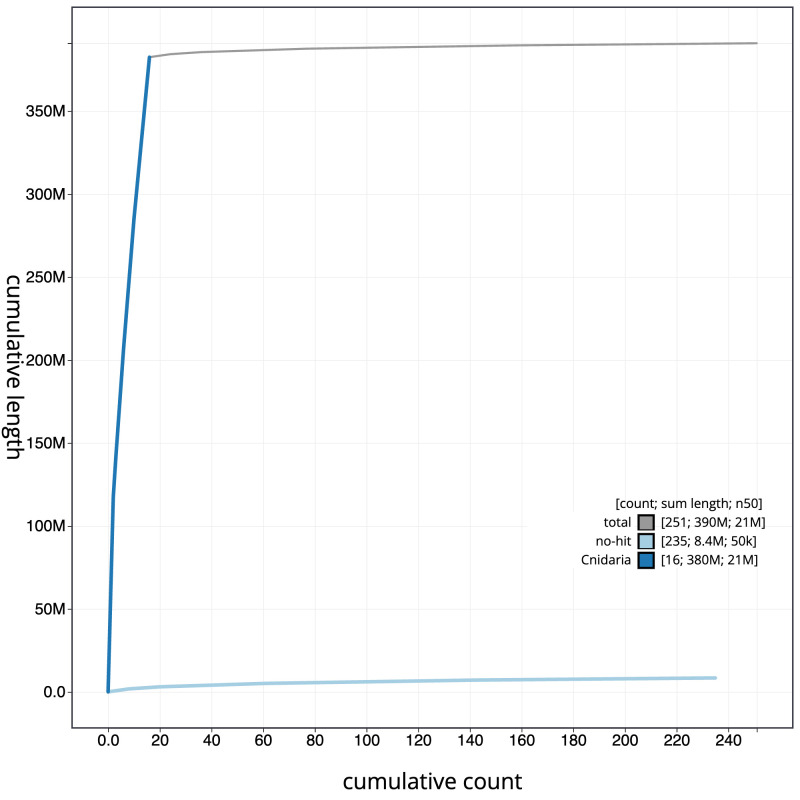
Genome assembly of *Metridium senile*, jaMetSeni4.1: BlobToolKit cumulative sequence plot. The grey line shows cumulative length for all scaffolds. Coloured lines show cumulative lengths of scaffolds assigned to each phylum using the buscogenes taxrule. An interactive version of this figure is available at https://blobtoolkit.genomehubs.org/view/Metridium%20senile/dataset/CATKSA01/cumulative.

**Figure 5.**
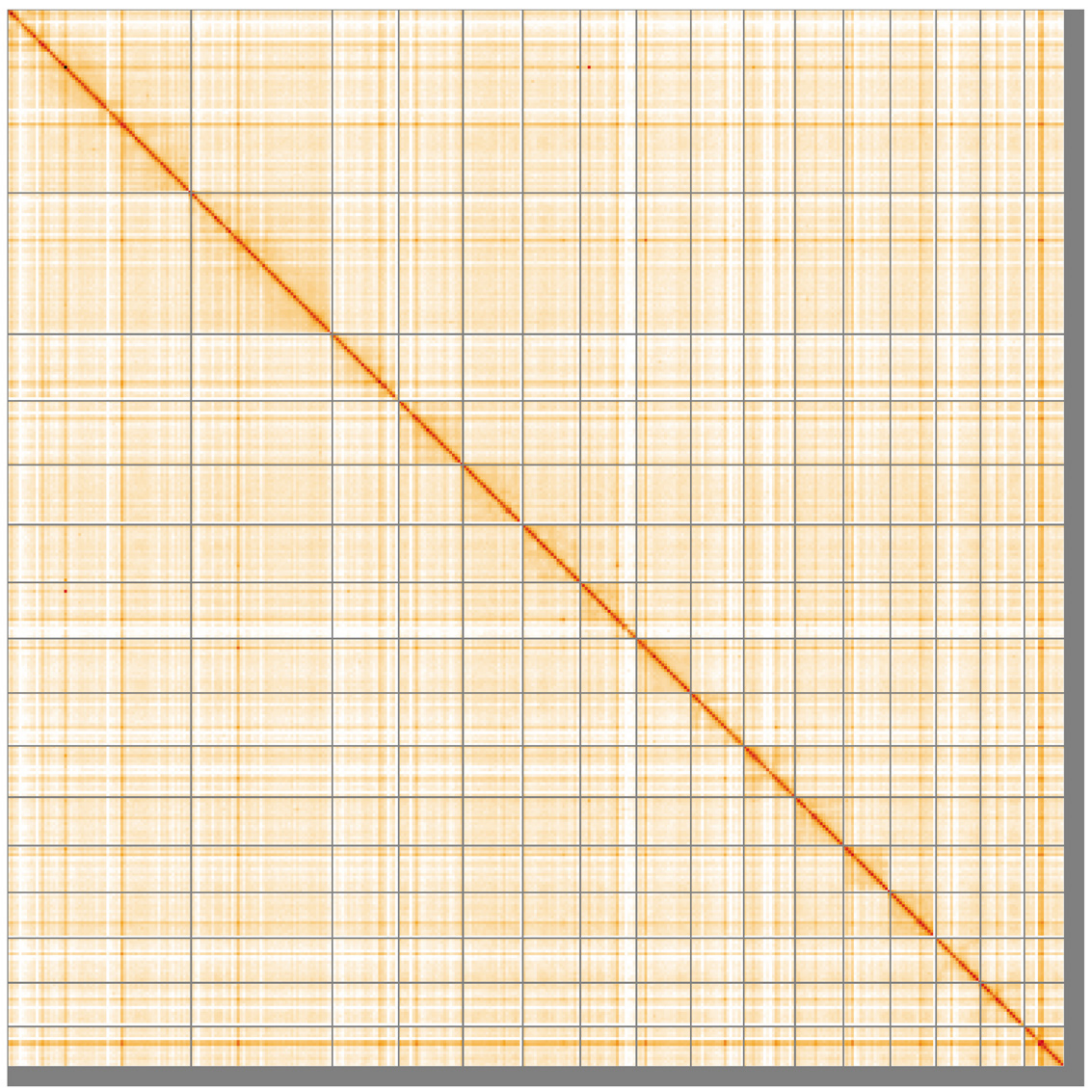
Genome assembly of *Metridium senile*, jaMetSeni4.1: Hi-C contact map of the jaMetSeni4.1 assembly, visualised using HiGlass. Chromosomes are shown in order of size from left to right and top to bottom. An interactive version of this figure may be viewed at https://genome-note-higlass.tol.sanger.ac.uk/l/?d=VIzq4JJXTx-ZKC8jiLPHTw.

**Table 2.** Chromosomal pseudomolecules in the genome assembly of *Metridium senile*, jaMetSeni4.

INSDC accession	Chromosome	Length (Mb)	GC%
OX459095.1	1	66.43	35.5
OX459096.1	2	51.09	35.0
OX459097.1	3	24.05	35.5
OX459098.1	4	23.19	35.5
OX459099.1	5	21.63	35.5
OX459100.1	6	20.83	35.5
OX459101.1	7	20.34	35.5
OX459102.1	8	19.63	35.0
OX459103.1	9	19.12	35.5
OX459104.1	10	18.58	35.0
OX459105.1	11	17.57	35.5
OX459106.1	12	16.92	35.5
OX459107.1	13	16.5	35.0
OX459108.1	14	16.13	35.5
OX459109.1	15	15.87	35.5
OX459110.1	16	14.63	35.0
OX459111.1	MT	0.02	38.0

The estimated Quality Value (QV) of the final assembly is 55 with *k*-mer completeness of 99.99%, and the assembly has a BUSCO v5.3.2 completeness of 96.6% (single = 96.0%, duplicated = 0.6%), using the metazoa_odb10 reference set ( *n* = 954).

Metadata for specimens, spectral estimates, sequencing runs, contaminants and pre-curation assembly statistics can be found at https://links.tol.sanger.ac.uk/species/6116.

## Methods

### Sample acquisition and nucleic acid extraction

A *Metridium senile* (specimen ID MBA-210326-002A, individual jaMetSeni4) was collected from Queen Anne's Battery Marina visitors’ pontoon, Plymouth, UK (latitude 50.36, longitude -4.13) on 2021-03-26. The specimen was collected by John Bishop and Patrick Adkins, and identified by Rob Mrowicki and Patrick Adkins (all Marine Biological Association), and then preserved in liquid nitrogen. The specimen used for Hi-C data (specimen ID SAN0000175, individual jaMetSeni1) was collected by Mark Blaxter (Wellcome Sanger Institute) in Great Cumbrae, Scotland (latitude 55.75, longitude –4.91) on 2020-08-24.

DNA was extracted at the Tree of Life laboratory, Wellcome Sanger Institute (WSI). The jaMetSeni4 sample was weighed and dissected on dry ice with tissue set aside for Hi-C sequencing. Bodywall tissue was cryogenically disrupted to a fine powder using a Covaris cryoPREP Automated Dry Pulveriser, receiving multiple impacts. High molecular weight (HMW) DNA was extracted using the Qiagen MagAttract HMW DNA extraction kit. Low molecular weight DNA was removed from a 20 ng aliquot of extracted DNA using the 0.8X AMpure XP purification kit prior to 10X Chromium sequencing; a minimum of 50 ng DNA was submitted for 10X sequencing. HMW DNA was sheared into an average fragment size of 12–20 kb in a Megaruptor 3 system with speed setting 30. Sheared DNA was purified by solid-phase reversible immobilisation using AMPure PB beads with a 1.8X ratio of beads to sample to remove the shorter fragments and concentrate the DNA sample. The concentration of the sheared and purified DNA was assessed using a Nanodrop spectrophotometer and Qubit Fluorometer and Qubit dsDNA High Sensitivity Assay kit. Fragment size distribution was evaluated by running the sample on the FemtoPulse system.

Protocols employed by the Tree of Life laboratory are publicly available on protocols.io: https://dx.doi.org/10.17504/protocols.io.8epv5xxy6g1b/v1.

### Sequencing

Pacific Biosciences HiFi circular consensus DNA sequencing libraries were constructed according to the manufacturers’ instructions. DNA sequencing was performed by the Scientific Operations core at the WSI on a Pacific Biosciences SEQUEL II (HiFi) instrument. Hi-C data were generated from tissue of jaMetSeni1 using the Arima2 kit and sequenced on the Illumina NovaSeq 6000 instrument.

### Genome assembly, curation and evaluation

Assembly was carried out with Hifiasm ( Cheng *et al.*, 2021) and haplotypic duplication was identified and removed with purge_dups ( Guan *et al.*, 2020). The assembly was then scaffolded with Hi-C data ( Rao *et al.*, 2014) using YaHS ( Zhou *et al.*, 2023). The assembly was checked for contamination and corrected as described previously ( Howe *et al.*, 2021). Manual curation was performed using HiGlass ( Kerpedjiev *et al.*, 2018) and Pretext ( Harry, 2022). The mitochondrial genome was assembled using MitoHiFi ( Uliano-Silva *et al.*, 2023), which runs MitoFinder ( Allio *et al.*, 2020) or MITOS ( Bernt *et al.*, 2013) and uses these annotations to select the final mitochondrial contig and to ensure the general quality of the sequence.

A Hi-C map for the final assembly was produced using bwa-mem2 ( Vasimuddin *et al.*, 2019) in the Cooler file format ( Abdennur & Mirny, 2020). To assess the assembly metrics, the *k*-mer completeness and QV consensus quality values were calculated in Merqury ( Rhie *et al.*, 2020). This work was done using Nextflow ( Di Tommaso *et al.*, 2017) DSL2 pipelines “sanger-tol/readmapping” ( Surana *et al.*, 2023a) and “sanger-tol/genomenote” ( Surana *et al.*, 2023b). The genome was analysed within the BlobToolKit environment ( Challis *et al.*, 2020) and BUSCO scores ( Manni *et al.*, 2021; Simão *et al.*, 2015) were calculated.

Table 3 contains a list of relevant software tool versions and sources.

**Table 3.** Software tools: versions and sources.

Software tool	Version	Source
BlobToolKit	4.1.7	https://github.com/blobtoolkit/blobtoolkit
BUSCO	5.3.2	https://gitlab.com/ezlab/busco
Hifiasm	0.16.1-r375	https://github.com/chhylp123/hifiasm
HiGlass	1.11.6	https://github.com/higlass/higlass
Merqury	MerquryFK	https://github.com/thegenemyers/MERQURY.FK
MitoHiFi	2	https://github.com/marcelauliano/MitoHiFi
PretextView	0.2	https://github.com/wtsi-hpag/PretextView
purge_dups	1.2.3	https://github.com/dfguan/purge_dups
sanger-tol/genomenote	v1.0	https://github.com/sanger-tol/genomenote
sanger-tol/readmapping	1.1.0	https://github.com/sanger-tol/readmapping/tree/1.1.0
YaHS	1.2a	https://github.com/c-zhou/yahs

### Wellcome Sanger Institute – Legal and Governance

The materials that have contributed to this genome note have been supplied by a Darwin Tree of Life Partner. The submission of materials by a Darwin Tree of Life Partner is subject to the **‘Darwin Tree of Life Project Sampling Code of Practice’**, which can be found in full on the Darwin Tree of Life website here. By agreeing with and signing up to the Sampling Code of Practice, the Darwin Tree of Life Partner agrees they will meet the legal and ethical requirements and standards set out within this document in respect of all samples acquired for, and supplied to, the Darwin Tree of Life Project. 

Further, the Wellcome Sanger Institute employs a process whereby due diligence is carried out proportionate to the nature of the materials themselves, and the circumstances under which they have been/are to be collected and provided for use. The purpose of this is to address and mitigate any potential legal and/or ethical implications of receipt and use of the materials as part of the research project, and to ensure that in doing so we align with best practice wherever possible. The overarching areas of consideration are:

• Ethical review of provenance and sourcing of the material

• Legality of collection, transfer and use (national and international) 

Each transfer of samples is further undertaken according to a Research Collaboration Agreement or Material Transfer Agreement entered into by the Darwin Tree of Life Partner, Genome Research Limited (operating as the Wellcome Sanger Institute), and in some circumstances other Darwin Tree of Life collaborators.

## Data Availability

European Nucleotide Archive: *Metridium senile* (brown sea anemone). Accession number PRJEB59155; https://identifiers.org/ena.embl/PRJEB59155 ( Wellcome Sanger Institute, 2023). The genome sequence is released openly for reuse. The *Metridium senile* genome sequencing initiative is part of the Darwin Tree of Life (DToL) project. All raw sequence data and the assembly have been deposited in INSDC databases. The genome will be annotated using available RNA-Seq data and presented through the Ensembl pipeline at the European Bioinformatics Institute. Raw data and assembly accession identifiers are reported in Table 1.

## Author information

Members of the Marine Biological Association Genome Acquisition Lab are listed here: https://doi.org/10.5281/zenodo.4783604.

Members of the Darwin Tree of Life Barcoding collective are listed here: https://doi.org/10.5281/zenodo.4893703.

Members of the Wellcome Sanger Institute Tree of Life programme are listed here: https://doi.org/10.5281/zenodo.4783585.

Members of Wellcome Sanger Institute Scientific Operations: DNA Pipelines collective are listed here: https://doi.org/10.5281/zenodo.4790455.

Members of the Tree of Life Core Informatics collective are listed here: https://doi.org/10.5281/zenodo.5013541.

Members of the Darwin Tree of Life Consortium are listed here: https://doi.org/10.5281/zenodo.4783558.
