# Supplementary material for: The genome sequence of the brown sea anemone, Metridium senile (Linnaeus, 1761)
Source: Wellcome Open Res. 2023 Nov 20;8:536. [Version 1] doi: 10.12688/wellcomeopenres.20376.1 (PMC11362738; doi:10.12688/wellcomeopenres.20376.1)
Supplement: Peer review history

# Reviewer report: Reviewer response for version 1

**DOI:** 10.21956/wellcomeopenres.22563.r73514

**Published:** 2024-08-29

**Authors:** Peter Prentis

**Affiliations:** 1 Centre for Agriculture and Bioeconomy and School of Biology and Environmental Sciences, Faculty of Science, Queensland University of Technology, Brisbane, Queensland, Australia

This report is fine for Indexing in its current form. The rationale for the research is well explained, the methods for the genome assembly has sufficient detail and the results are easy follow and logically set out. It will provide an excellent resource for the sea anemone, coral and Cnidaria community.

Are sufficient details of methods and materials provided to allow replication by others?

Yes

Is the rationale for creating the dataset(s) clearly described?

Yes

Are the datasets clearly presented in a useable and accessible format?

Yes

Are the protocols appropriate and is the work technically sound?

Yes

Reviewer Expertise:

Cnidarian genomics

I confirm that I have read this submission and believe that I have an appropriate level of expertise to confirm that it is of an acceptable scientific standard.

# Reviewer report: Reviewer response for version 1

**DOI:** 10.21956/wellcomeopenres.22563.r76167

**Published:** 2024-04-17

**Authors:** Christian Voolstra

**Affiliations:** 1 Department of Biology, University of Konstanz, Konstanz, Germany

The manuscript is a rather brief genome report on the sequencing and assembly of the *Metridium senile *genome;

 The background section lists rather old studies; it would be nice to add some more recent references for the last sentence/statement in the background section;

 For the Methods, it would be nice to provide a GitHub repo or similar that actually describe the detailed code used; for instance, the manual assembly curation is very brief: how were haplotypic duplications identified and removed?

 The data availability section states the Project webpage, but the actual genome sequence is not download-able? Also, the genome is not annotated. I suppose this comes at a later stage, or there is an automated EMBL pipeline? but something to this regard should be stated to inform the interested researcher.

Are sufficient details of methods and materials provided to allow replication by others?

Yes

Is the rationale for creating the dataset(s) clearly described?

Yes

Are the datasets clearly presented in a useable and accessible format?

Yes

Are the protocols appropriate and is the work technically sound?

Yes

Reviewer Expertise:

cnidarian/bacterial genome/transcriptome analysis

I confirm that I have read this submission and believe that I have an appropriate level of expertise to confirm that it is of an acceptable scientific standard, however I have significant reservations, as outlined above.

# Reviewer report: Reviewer response for version 1

**DOI:** 10.21956/wellcomeopenres.22563.r73513

**Published:** 2024-02-27

**Authors:** Steinar Daae Johansen

**Affiliations:** 1 Nord University, Bodø, Norway

1. The current data note is well presented. The introduction gives an overview of taxonomic issues, and explains well the current status of Metridium senile sub-species. The DNA sequencing, assembly, and feature analyses follow the current practice at the "Darwin Tree of Life" consortium. 
2. The ca 391 Mb genome was found to be assigned to 16 chromosomal-like scaffolds. The total number of scaffolds (250) could have been significantly reduced if the authors, in addition to PacBio and Hi-C, also have applied Oxford Nanopore long-reads and ultra long-reads. Great technical improvements have been made on ON sequencing the last couple of years. Anyway, ON is in my view not mandatory for this data note, but hopefully the consortium will consider this technology in future genome reports. 

Are sufficient details of methods and materials provided to allow replication by others?

Yes

Is the rationale for creating the dataset(s) clearly described?

Yes

Are the datasets clearly presented in a useable and accessible format?

Yes

Are the protocols appropriate and is the work technically sound?

Yes

Reviewer Expertise:

Eukaryote genome sequencing, mitogenomics, RNA biology

I confirm that I have read this submission and believe that I have an appropriate level of expertise to confirm that it is of an acceptable scientific standard.
